# Reliability and Repeatability Analysis of Indices to Measure Gait Deterioration in MS Patients during Prolonged Walking

**DOI:** 10.3390/s20185063

**Published:** 2020-09-06

**Authors:** Juri Taborri, Valeria Studer, Paola Grossi, Laura Brambilla, Fabrizio Patanè, Maria Teresa Ferrò, Renato Mantegazza, Stefano Rossi

**Affiliations:** 1 Department of Economics, Engineering, Society and Business Organization (DEIM), University of Tuscia, 01100 Viterbo, Italy; stefano.rossi@unitus.it; 2 Multiple Sclerosis Center, Neurology Department, Martini Hospital, 10121 Turin, Italy; valeria.studer@virgilio.it; 3 Neuroimmunology Center for Multiple Sclerosis, Cerebrovascular Department, ASST Crema, 52242 Crema, Italy; paolagrossi76@hotmail.com (P.G.); maraferro@libero.it (M.T.F.); 4 Operating Units of Autoimmune and Neuromuscular Diseases, IRCCS Foundation Neurological Institute Carlo Besta, 20133 Milano, Italy; laura.brambilla@istituto-besta.it (L.B.); renato.mantegazza@istituto-besta.it (R.M.); 5 Department of Engineering, Mech. Meas. and Microelectr.lab (M3lab), University Niccolò Cusano, 00166 Rome, Italy; fabrizio.patane@unicusano.it

**Keywords:** reliability, repeatability, synthetic indices, multiple sclerosis, 6-min walking test, fatigue

## Abstract

Gait deterioration caused by prolonged walking represents one of the main consequences of multiple sclerosis (MS). This study aims at proposing quantitative indices to measure the gait deterioration effects. The experimental protocol consisted in a 6-min walking test and it involved nine patients with MS and twenty-six healthy subjects. Pathology severity was assessed through the Expanded Disability Status Scale. Seven inertial units were used to gather lower limb kinematics. Gait variability and asymmetry were assessed by coefficient of variation (CoV) and symmetry index (SI), respectively. The evolution of ROM (range of motion), CoV, and SI was computed analyzing data divided into six 60-s subgroups. Maximum difference among subgroups and the difference between the first minute and the remaining five were computed. The indices were analyzed for intra- and inter-day reliability and repeatability. Correlation with clinical scores was also evaluated. Good to excellent reliability was found for all indices. The computed standard deviations allowed us to affirm the good repeatability of the indices. The outcomes suggested walking-related fatigue leads to an always more variable kinematics in MS, in terms of changes in ROM, increase of variability and asymmetry. The hip asymmetry strongly correlated with the clinical disability.

## 1. Introduction

Multiple sclerosis (MS) is one of the main neurological diseases arising in early to middle adulthood with a prevalence of 2.5 million in the world [1]. MS is a chronic autoimmune inflammatory disease of the central nervous system that causes demyelination and neuronal loss. As a consequence, MS clinical disability is mainly driven by cognitive and motor deficits [2]; where, locomotion results to be one of the main affected activities leading to a worsening of life quality. Muscle weakness, ataxia, and muscle spasms are the most common motor symptoms [3,4]. It is worth noticing that 85% of MS patients report gait disturbance as their main compliant [5]. Several studies showed that gait speed, gait asymmetry, gait variability, and fatigue are the main differences when comparing MS patients with healthy subjects [6,7]. Actually, the most widespread methodology for assessing the clinical disability is the administration of clinical scales, such as the Expanded Disability Status Scale (EDSS) [8]. Nevertheless, the reliability of this scale and its capability to monitor the progression of the pathology is often questioned; in addition, it is not focused only on motor disturbance, demonstrating that the objective and repeatable assessment of gait deterioration remains challenging [9]. Gait deterioration caused by prolonged walking is the main consequence of fatigue. Central fatigue in MS is associated with both neurotransmitters disturbance and circadian rhythm disorders; in addition, it has been demonstrated as the perception of fatigue may arise since an early stage of the disease [10]. In addition, it is well-known that the quantification of fatigue effects is challenging, mainly due to the two main independent aspects, i.e., the perception of fatigue and the performance fatigability [11]. Kluger et al. explained the strong difference between the two aspects, suggesting the identification of specific measures to separately address fatigue and fatigability [11]. Actually, different rating scales have been proposed for the quantification of fatigue; among them, the Fatigue Scale for Motor and Cognition (FSMC) is the most applied [12]. However, two main issues are raised from the analysis of the scale. Firstly, the scale is questionnaire-based, causing a subjectivity of the answers and, then, no information about the motor aspect is taken into account [13]. To overcome these limitations, laboratory gait analysis performed by means of motion capture systems still represents the gold standard to fully understand the mechanism of gait deterioration in MS patients [14]; however, in the last decades, inertial units reached a great popularity thanks to their wearability, low cost, and portability [15,16,17,18]. As well, the use of virtual reality for gait parameter assessment has also received great popularity [19].

Focusing on patients with MS, McGinnis et al. proposed a machine-learning approach fed with inertial data to estimate gait speed in an outdoor environment [20] and Alaqtash and colleagues evaluated the possibility of using a fuzzy computation algorithm for the extraction of gait features on inertial data to assess their gait mobility [21]. Several studies have been already proposed in literature aiming at providing objective measures of fatigue using both optoelectronic systems and inertial units. Sehle et al. reported differences in step length, step width, and knee flexion when comparing MS patients and healthy subjects in performing a walking test on a treadmill until the perception of fatigue [13]. A one-minute walking test was also used to propose synthetic indices based on range of motion and variability able to distinguish between MS patients and healthy subjects [22]. Although a set of walking tests is available for quantifying fatigue, most of the studies elected the 6-min walking test (6MWT) as the most suitable for the assessment of walking-related fatigue [23]. Leone et al. evaluated the difference related to the distance covered during the first and the last minute of the test, promoting this index as useful for depicting the walking-related fatigue [24]. McLoughlin and colleagues assessed effects of fatigue due to the execution of 6MWT on kinematics and kinetics parameters by performing a gait analysis before and after the 6MWT, finding differences on ankle dorsiflexion, knee and hip flexor moment, and no variations related to the spatio-temporal parameters [25].

All the above-mentioned studies were focused on evaluating the effects of prolonged walking, not during the execution of the task, but by comparing data obtained before and after the task; thus, no studies evaluated the effects of fatigue and their evolution on gait kinematics during the execution of the 6MWT. It is worth noticing that only the preliminary study proposed by the same author of this paper demonstrated that the evolution of the range of motion related to the lower limb joints could be considered as a suitable analysis for the objective assessment of gait variability and fatigue in MS patients [26]. In addition, no studies took into account the analysis of walking-related fatigue effects on gait variability and gait asymmetry, even though it is well known that these two factors represent two of the main consequences of neurological diseases, leading to an increase of fall risk. Finally, all the indices proposed in the previous studies extracted from the analysis of a 6MWT, with the exception of the distance covered during the task, have not been tested for reliability and repeatability, even if these two metrological characteristics are recognized as fundamental in order to enhance the use of objective indices in clinical settings [27].

From this perspective, this study aims at proposing and validating reliable and repeatable synthetic indices based on range of motion, gait variability, and gait asymmetry for the quantification of walking deterioration in MS patients when performing prolonged walking, i.e., the application of 6MWT, in comparison with age-matched healthy subjects. As a further aim, we want to assess the correlation between these indices and the EDSS score. These indices will be able to distinguish MS patients from healthy subjects and to evaluate the pathology severity, leading to a more specific design of pathology treatments.

## 2. Materials and Methods

### 2.1. Participants

Five healthy subjects (mean age 45 ± 2 years) were recruited for reliability and repeatability analysis. Subjects were included if they did not have pathologies affecting motor functions and subjects who have undergone orthopaedical surgery in the last six months were excluded.

Two further cohorts of subjects were enrolled in the study. The first one, named as MS group, was composed of nine patients affected by relapsing-remitting multiple sclerosis (mean age 45 ± 5); while the second, named as the control group (CG), was composed of twenty-six age-matched healthy subjects (45 ± 9). Patients were selected at the Neurological Institute “Carlo Besta” of Milan and at the Neuroimmunology Center of ASST of Crema and they were included in the study if they met the following inclusion criteria: feasibility to walk without external aid and EDSS score ranged from 1 to 4 determined by a skilled neurologist within the last month. Demographical details of patients are reported in Table 1. All participants were initially informed about the aim of the study and they signed a written consent. Experimental procedures were in accordance with the principles declaimed in the Declaration of Helsinki and they were approved by the Ethical Committees of the Neurological Institute “Carlo Besta” of Milan (EC record n. 43, 6 September 2017) and ASST of Crema (n. 443, 14 December 2017).

### 2.2. Instrumentation

Commercially available inertial measurement units (IMUs) were used to gather the kinematics of lower limbs of the participants. More specifically, the Xsens MTws produced by Xsens Technologies (Enschede, The Netherland) were selected among others. Each unit is composed of a tri-axial linear accelerometer, a tri-axial gyroscope, and a tri-axial magnetometer. Further details on sensor specifications can be found in [28]. Several studies have already been performed to evaluate the performance of Xsens’ IMUs with respect to the optoelectronic system, which still remains the gold standard in gait analysis [29]. Xsens showed an accuracy in the estimation of joint angles lower than 1° for the knee and ankle joints and equal to 1° for the hip one [30]. It is well demonstrated that the effects of magnetic field distortions can be observed mainly in the out-of-sagittal planes, which are the less involved in walking, and the joint angles evaluated in the sagittal plane are repeatable both in indoor and outdoor environments [31]. Moreover, using a validated functional calibration, the lower limb joint angles are not influenced by the sensor positions and inclinations on the body segments [32]. Finally, the low mass of each IMU (16 g) guarantees to not influence the gait patterns of the participants. Thanks to the reported metrological characteristics, Xsens’ IMUs represent the most widespread wearable sensors to perform gait analysis, as also reported in [33].

### 2.3. Experimental Procedure

**Figure 1:**
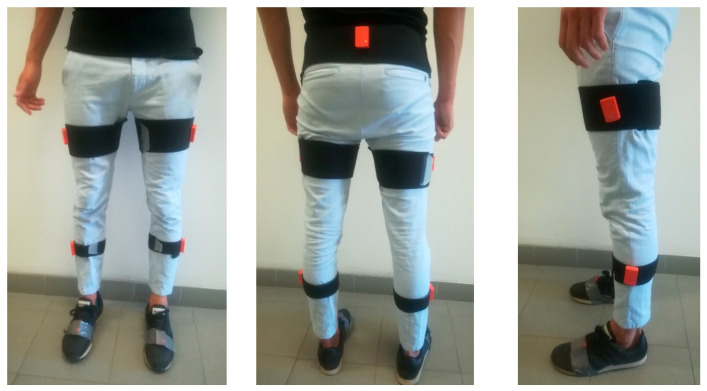
Example of IMU (inertial measurement unit) placement on lower limbs of one subject: frontal, back, and lateral view.

Seven IMUs were placed by used ad-hoc elastic belts on pelvis, thigh, shank, and foot of both lower limbs (Figure 1). Each participant, before starting the experimental protocol, performed several strides until he/she felt familiar with the equipment to avoid gait pattern alterations due to the presence of the IMUs.

The experimental protocol was subdivided into two sub-protocols: the former, addressed as *reliability and repeatability protocol*, was focused on the quantification of the reliability and repeatability of the herewith-proposed novel indices; the latter, addressed as *evaluation protocol*, was performed to analyze the fatigue effects on gait kinematics of subjects with MS when performing prolonged walking. Both protocols consisted in the execution of a 6-min walking test (6MWT). Participants were asked to walk at a self-selected speed along a leveled pathway of 15 m continuously for six minutes with their preferred comfortable shoes. Subjects were instructed to select the speed in order to cover the maximum distance possible during the six minutes and to walk forth and back within two turn lines distant 15 m on the floor. They had to reverse their walking direction by 180°, performing a curve at each turn line. Participants were free to choose the direction of the turn; however, in order to avoid dizziness, it was suggested they perform the turn alternatively through the right and left shoulder. In addition, an operator informed participants as each minute went by and participants could decide to stop their walking to rest during the execution of the test [34]. All participants were able to complete the entire protocol and no one performed a rest during the execution. The entire protocol, also considering the time spent in the sensorization of the subject, lasted approximately 15 min per participant. It is worth noticing that before the execution of the 6MWT, IMU data were recorded by asking participants to keep still in two positions: a standing upright posture and a sitting position with the trunk backward inclined and the legs extended with heels touching the ground. These two positions were used for the functional calibration of the sensors [32].

For the reliability and repeatability protocol, the five healthy subjects repeated the 6MWT five times in five different days and, for each day, two repetitions were performed. In particular, the two repetitions in the same day were used to evaluate the intra-day reliability and the intra-day repeatability. Each 6MWT was repeated at a distance of 15 min without removing the sensors attached on the lower limb; the functional calibration was performed before each repetition. The repetitions in five different days were used to assess the inter-day reliability and the inter-day repeatability. Thus, the intra-day analysis allowed quantifying the robustness of the here proposed synthetic indices with respect to the intrinsic gait variability; while the inter-day analysis permitted to assess the effects induced by the re-placement of the IMUs on body segments and the daily changes of individual gait patterns [35]. Conversely, only a repetition of the 6MWT was performed by MS and CG groups during the evaluation protocol.

### 2.4. Data Processing and Analysis

Distance covered during the fixed time was computed for each participant by counting the number of turns during the trial and adding the meters covered within the end of the trial after the last turn. The meters to add were computed by manually measuring the distance where participant stopped his/her walking. This index is named walking length (WL). Then, the average gait speed was found, dividing the WL by the fixed time of six minutes.

Functional calibration proposed by Palermo et al. was used to extract angle curves related to the hip, knee, and ankle joint of both sides thanks to the application of a biomechanical model [32]. Successively, angle curves were divided into each gait cycle by applying an already proposed threshold-based algorithm [36]. Specifically, the algorithm is based on the analysis of the angular velocity of the shank and it consists firstly in the identification of the maximum of the curve and then in the selection of the minimum that anticipates the maximum; the found minimum represents the heel strike. Stride is, thus, defined as the time distance between two consecutive heel strikes [37,38]. The algorithm was applied separately for right and left side. Then, angle curves related to each stride were normalized at 100 samples. Before computing synthetic indices based on kinematic data, angle curves related to turning phases and to the two strides after and before each turn were excluded from the successive analyses in order to avoid possible turning effects on lower limb kinematics. In addition, the first three strides of the task were excluded to avoid the influence of the acceleration phase [26]. After data selection, angle curves were divided into six time subgroups. Each group contained data related to 60 s. It is worth highlighting that the first time subgroup did not include the first three strides due to the data reduction performed, as previously mentioned. This division allowed evaluating the evolution of gait kinematics minute by minute during the prolonged walking. Then, range of motion (ROM) of joint angles in the sagittal plane was computed for each stride; means mROM and standard deviations sdROM were calculated by considering together ROM related to the strides within the same time subgroup. This methodology led to obtain six values of mROM for each participant, each joint and each side. In order to quantify the gait variability during the execution of the test, the coefficient of variation (CoV) of ROM was computed for each time subgroup, individually for each participant, each joint and each side, as follows:

(1) $$\mathrm{CoV}=\;\frac{\mathrm{sdROM}}{\mathrm{mROM}}\;\times 100.$$

Greater values of the CoV correspond to a greater variability of the kinematic data.

To assess the gait asymmetry, SI_i_ was computed for each time subgroup, individually for each participant and each joint, following the equation [22]:

(2) $${\mathrm{SI}}_{i}=\;\frac{\left|{\;\mathrm{mROM}}_{\mathrm{ri}}-{\;\mathrm{mROM}}_{\mathrm{li}}\right|}{\frac{1}{2}\left({\mathrm{mROM}}_{\mathrm{ri}}+{\;\mathrm{mROM}}_{\mathrm{li}}\right)}\;\times 100,$$

where right and left limbs have been addressed as *r* and *l* and *i* can be h (hip), k (knee), and a (ankle). This index allows to understand how a prolonged walking influences the gait symmetry in patients with MS and it can assume value from 0 to 200%, considering that greater values correspond to a greater asymmetry.

Finally, to understand how the prolonged walking affects the kinematics, relative decrements for the mROM were evaluated in order to take into account the initial differences among subjects:

(3) $$\delta =\;\frac{ROMi-\;ROM_{1}}{ROM_{1}}\times 100,$$

where $mROM_{1}$ is computed at the first time subgroup and mROMi is computed at each of the other time subgroups. Instead for CoV and SI, the absolute decrements δ between the first minute and each one of the remaining five were computed, since they are already normalized for each subject and expressed as percentage. The indices were, then, named as $\delta_{1-2}^{}$, $\delta_{1-3}^{}$, $\delta_{1-4}^{},\;\delta_{1-5}^{}$, and $\delta_{1-6}^{}$, where 1 to 6 are the number of the time subgroups. Greater values of the decrement δ can be associated with a greater variability of the indices during the execution of the task, consequently, a deterioration in the gait patterns [25]. In addition, as a synthetic index to resume all the previous findings, for all the computed parameters, the absolute value of the maximum difference among time subgroups was computed, addressed as Δ_max_. This index permitted to assess the maximum variability of participants’ kinematics during the entire 6MWT. Specifically, the highest is the value, the highest the maximum variability.

### 2.5. Reliability and Repeatability Analysis

As regarding the reliability, to assess the intra-day reliability, inter-class correlation coefficient (ICC) was computed for all the examined indices individually for each of the five sessions. Then, the range of ICC values was found for each parameter. To assess the inter-day reliability, the mean value of each parameter was firstly computed considering the two repetitions within the same day and then the ICC values were computed for each parameter. ICC values in the range 0.00–0.39 were classified as poor, 0.40–0.59 as fair, 0.60–0.74 as good, and 0.75–1.00 as excellent [39].

Focusing on the repeatability, standard deviations (SD) of each index were computed considering the repetitions in the same day for each subject; then, the maximum value of SD across subjects and days was reported to quantify the intra-day repeatability. Successively, SDs of each index were computed for each subject, considering the five days and the maximum value across subjects was reported to quantify the inter-day repeatability.

### 2.6. Statistical Analysis

Considering the evaluation protocol, all data were tested for normality through the Saphiro–Walk test. In order to compare the two examined groups, we selected only the right side for CG since low values of SI were found and, for each subject with MS, we considered separately the most and the least variable side identified as the ones associated with the greatest and the lowest value of Δ_max_, respectively.

Successively, two steps of statistical analyses were conducted. Firstly, an independent *t*-test was applied to verify if the distance covered during the execution of the 6MWT depended on the two groups, i.e., MS and CG; the same test was also applied on data related to the relative and absolute decrements and Δmax to understand if statistical differences occurred among CG and the most variable lower limb side of subjects with MS, considering each index independently. Secondly, by only focusing on the MS group, a two-way repeated measures ANOVA was applied to decrements related to mROM and CoV in order to assess the effects due to the time subgroups and the body side. If the interaction between the main effects occurs, one-way repeated ANOVA was applied independently on body sides and time subgroups. As concerning the decrements related to the SI, a one-way repeated measures ANOVA was performed with time subgroups as the main effect. In all repeated measure tests, when the assumption of sphericity was violated, the Greenhouse–Geisser correction was considered. In case of statistical differences, a Bonferroni test for multiple comparisons was applied. For all the performed statistical tests, a significance level equal to 0.05 was considered.

Finally, we performed Spearman’s test to assess the correlation between the EDSS and the here proposed synthetic indices. The r can be interpreted as: (i) no correlation, if |r| ≤ 0.1; (ii) mild/modest correlation, if 0.1 < |r| ≤ 0.3; (iii) moderate correlation, if 0.3 < |r| ≤ 0.6; (iv) strong correlation, if 0.6 < |r| < 1; and, finally, (v) perfect correlation, if |r| = 1. For this analysis, we considered only indices computed for the most variable lower limb side and multiple correlation correction was applied, according to the methodology reported in [40].

## 3. Results

### 3.1. Reliability and Repeatability Analysis

The range of the ICC values for the intra-day reliability related to the parameters computed for the 6MWT is reported in Table 2.

The ICC values related to the Δ_max_ index were always within the range of excellent reliability for all joints and all parameters, as well as the values for the WL. Conversely, the values related to the decrement δ ranged from good to excellent reliability; however, they were always equal or greater than 0.70. More specifically, indices related to mROM ranged from 0.70 to 0.83 for the hip joint, from 0.70 to 0.87 for the knee joint, and from 0.70 to 0.81 for the ankle joint. ICC computed for the indices related to the CoV ranged from 0.70 to 0.94 for the hip, from 0.71 to 0.95 for the knee, and from 0.73 to 0.94 for the ankle. Finally, the ICC for the SI ranged from 0.70 to 0.91 for the hip, from 0.70 to 0.89 for the knee, and from 0.70 to 0.91 for the ankle.

ICC values for the inter-day reliability related to the parameters computed for the 6MWT are reported in Table 3.

By analyzing the results, we can assess that the ICC values related to the Δ_max_ index were always within the range of excellent reliability for all joints and all parameters, as well as the values for the WL. Conversely, ICC values for the δ related to the mROM showed from good to excellent reliability, ranging from 0.71 to 0.78, considering all joints. The same range of reliability was observed also for the δ related to the SI; while an excellent reliability was found for all the δ related to the CoV.

Intra- and inter-day repeatability results are reported in Table 4. As concerning the intra-day, the lowest values of SD were always associated to the Δ_max_ for all the examined indices and values were always lower than 0.8°, 0.5%, and 0.8% for mROM, CoV, and SI, respectively. SD values for the δ related to the mROM showed values greater than 1.0°, especially concerning the knee joint. CoV showed low values of SD also when considering the δ always lower than 0.8%; while SD up to 1.2% was found for SI. By moving to the inter-day, we can assess that mROM showed lower value of SD for all joints if comparing with the intra-day. Similar behavior was related to SI; conversely increased values were obtained when looking at the inter-day repeatability of the CoV, up to 1.1%. WL showed similar SD value for the intra- and inter-day repeatability equal to 8.1 and 8.5 m, respectively.

### 3.2. Evaluation Protocol

**Figure 2:**
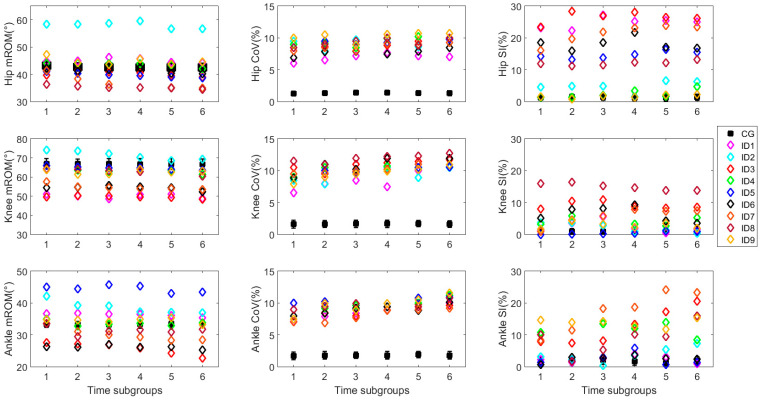
Evolution of mean range of motion (mROM), coefficient of variation (CoV) and symmetry index (SI) related to the six time subgroups for each joint. Black square and error bar represent mean value and standard deviation of control group. For the multiple sclerosis (MS) group, only the most variable side is reported.

Means and standard deviations of the WL are reported in Table 5, as well as the average gait speed. Statistical analysis revealed difference in meters covered during the execution of the test considering the two examined groups. The evolution of the mROM, CoV, and SI during the execution of the 6MWT for all joints are reported in Figure 2. As regarding the mROM, we can observe that patients affected by MS generally showed a reduction of the ROM related to the hip and the knee joints, with the exception of the ID2. More specifically, the reduction was up to 12.0° for the hip joint and 15.0° for the knee joint. As regarding the ankle joint, a heterogeneous trend can be detected among subjects with MS; in fact, only four subjects showed lower ROM values than CG; while, the ID7 showed patterns similar to the CG and the remaining four greater ROM values.

Relative decrements for the mROM and absolute decrement for CoV and SI related to the control group (CG) and the most (MS+) variable side of MS group are reported in Table 6, as well, the results related to the Δ_max_. By analyzing the $\delta_{1-2}^{r}$, it can be observed for all joints a similar trend, which is a continuous increment of the indices as the minute passes for the MS patients. Statistical analysis showed differences between the most variable side of MS and CG from the fifth minute of the test regarding the hip joint (*p*-value = 0.01), from the third minute for the knee joint (*p*-value ranged from <0.01 to 0.02), and from the second minute for the ankle joint (*p*-value ranged from <0.01 to 0.04). As regarding Δ_max_, the values related to the most variable side of MS were always different from the ones related to CG for all joints with a *p*-value always lower than 0.01.

As regarding the CoV, greater values of ROM variability were found for MS. In particular, values up to 10.7%, 12.7%, and 11.6% were found for the hip, knee, and ankle joint, respectively; while in the CG group, the same indices reached value up to 1.4%, 1.7%, and 1.9%. In addition, a consistent inter-subject variability can be observed in the MS group, in fact, a variation above 5% was found among subjects. By analyzing the δ, an always greater gait variability was found in MS during the evolution of the walking test, reaching a variation up to 2.3% for the knee joint. Conversely, the CG group showed a variation of the gait variability always lower than 0.5%. Statistical analysis showed difference among the CG group and MS from the second minute regarding the hip joint (*p*-value ranged from <0.01 to 0.03). Concerning the knee joint, CG group was always statistically different from the parameters computed in the most variable side of MS (*p*-value < 0.01). By moving to the ankle joint, differences between groups were observed only during the last two minutes of the test (*p*-value = 0.01). Considering the Δ_max_, differences were found among values related to the CG and MS with a *p*-value always lower than 0.01 for all joints.

As regarding SI, we can observe from Figure 2 that patients affected by MS generally showed a greater gait asymmetry in all examined joints. Specifically, a value of SI up to 28.3% was found with respect to the maximum value of 2.1% related to the CG for the hip joint; up to 16.2% vs. 1.4% for the knee joint; and, up to 24.2% vs. 2.1% in CG. In addition, a high inter-subject variability was found in the MS group; in fact, results ranged from 1.0% to 28.3% among subjects. By analyzing the δ, the trend was similar to the other parameters, assessing a constant increase of the gait asymmetry with MS when performing a prolonged walking, reaching a variation up to 4.9% for the ankle joint. Conversely, a variation of SI always lower than 1.6% was found for CG. Statistical differences between groups were found in the last minute, from the second minute and from the third minute for the hip, knee, and ankle joint respectively, with *p*-value ranging from <0.01 to 0.04. By moving to the Δ_max_, values were always different between the two examined groups (*p* < 0.01) for all joints.

The relative decrements for mROM and absolute decrements for CoV and SI related to the most and the least variable side for MS patients are reported on Table 7. No significant interaction among time subgroups and between body sides emerged for each tested parameter (*p*-value always greater than 0.2). Comparing the most and the least variable side of the MS patients, body sides showed statistical differences for the mROM relative decrement of the knee joint (*p* = 0.04) and for the CoV absolute decrement related to the hip (*p* = 0.05) and knee joint (*p* = 0.02). Taking into account the effect of the time subgroup, statistical differences were found for all the tested indices. As regarding mROM, time effects can be observed between the first three time subgroups and the last two for the hip and knee joint (*p*-value ranged from 0.01 to 0.04), and, between the first time subgroup and the last one (*p* < 0.01) for the ankle joint. As for the CoV, differences between the first two time subgroups and the last one were found for all the examined joints (*p*-value ranged from <0.01 to 0.02). By moving to the SI, no differences were found for the hip joint, instead the first time subgroup was different from the last two for both the knee and the ankle joint (*p*-value equal to <0.01 and 0.02 respectively).

The value of the correlation between EDSS and each parameter computed for the most variable side, along with the relative *p*-values, are reported in Table 8. Strong and significant correlation was found for both all the δ and Δ_max_ related to the SI for the hip joint (r ranged from 0.65 to 0.90), for the Δ_max_ related to the mROM of the hip joint (0.70), and Δ_max_ and $\delta_{1-4}^{}$ related to the SI of the ankle joint (0.70 and 0.89, respectively),. All other indices showed mild or poor and non-significant correlation with the EDSS score.

## 4. Discussion

Through the aim to measure walking-related fatigue effects on gait kinematics, we comparatively examined nine patients with MS and twenty-six healthy subjects when performing a 6-min walking test. The evolution of the ROM, CoV, and SI during the execution of the test was taken into account and reliability and repeatability analyses of the proposed synthetic indices were performed both intra- and inter-day.

### 4.1. Are the Proposed Synthetic Indices Reliable and Repeatable?

Results related to the reliability permit to assume that the here proposed indices express an excellent reliability, considering the walking distance WL and the Δ_max_ for all the examined joints. Conversely, results recommend particular attention to the variability of the indices when handling data from some specific time subgroups of the task even though a good reliability was found. The reliability results are similar to the ones related to gait analysis performed by optoelectronic systems [29]. The good and excellent reliability found for the intra-day analysis allows assessing that the selected indices are not influenced by the intra-subject variability of the gait. Furthermore, the reported inter-day reliability permits to consider negligible the effects of both the sensors re-placement on body segments and the intra-subject variability of gait performed in different days. These negligible effects can be ascribed to the application of the functional calibration that was properly designed to avoid bias on the results of gait analysis performed by IMU due to a different alignment of the sensors on the body segments [32].

By moving the repeatability analysis, all the found maximum values of SD are comparable with the intrinsic accuracy related to the used inertial sensors, i.e., lower than 1° [30] (see Figure 2), for both intra-day and inter-day analysis. These results allowed affirming that the three variability sources, which are intra-subject gait variability, the sensor re-placement, and the inter-day gait changes, do not influence the repeatability of the novel proposed indices. In fact, the obtained results can be likely associated to the sensor accuracy rather than to the three sources of variability above reported. By comparing the results for the intra- and inter-day repeatability related to indices based on mROM and SI, we can affirm that the values associated with the inter-day repeatability are always comparable with the sensors accuracy even though greater values were found with respect to the intra-day analysis. This result allows us to state that a single test in a single day should be suitable for the gait analysis evaluation. This finding is in line with the one reported in other studies focused on gait analysis [39,41].

In addition, the greater reliability and repeatability of Δ_max_ than the decrement δ can be due to the different mathematical definition of these indices; in fact, Δ_max_ considers only the maximum difference during the overall task, whereas δ considers the specific time subgroup sequence that can change among the subjects. The high value of reliability related to the WL, close to the perfection, i.e., ICC equal to 1, and the high repeatability, suggested by the low value of SD, permits affirming that healthy subjects choose always the same velocity capable to avoid fatigue when the time duration of the walking test is the same, confirming the excellent reliability and repeatability of the traditional outcome of the 6MWT [34].

As a conclusion, the here proposed parameters can be considered robust with respect to the typical intra-subject gait variability and any potential differences occurred when comparing healthy subjects with patients affected by MS can be ascribed to the pathology effects. It is worth highlighting that the reliability and repeatability of synthetic indices are fundamental requirements to introduce them into the clinical practice, even though this metrological aspect is often neglected in biomechanical studies.

### 4.2. Does Prolonged Walling Lead to Changes in Gait Kinematics?

Patients affected with MS showed different gait kinematics with respect to healthy subjects in terms of a general reduction of the ROM, a greater value of gait variability and gait asymmetry already from the first minute of the walking. These findings confirmed that gait of MS patients differs from the control group also in short walking [42]. These findings may be the consequence of a motor control deficit, as reported in [43]. By concerning the gait variability, patients with MS showed more than twice as much variability in gait performance also during prolonged walking, confirming the results reported in [44] regarding the gait variability during one minute of walking. Several studies have demonstrated that the gait variability is strictly related to the gait velocity in patients affected by neurological disease; in particular, greater variability at hip, knee, and ankle level was observed in correspondence with a decrease of walking speed [45,46]. Thus, the here found differences in variability may be attributed to the smaller value of speed typical of subjects with MS to complete the motor task. Focusing on the gait asymmetry, we can assess that the degenerative changes caused by the pathology take place not equally on lower limb sides, in accordance with [47]. This finding suggests that walking of subjects with MS is characterized by a diminishing inter-limb coordination, reflecting also in a greater variability of movement patterns [13]. In addition, the overall inter-subject variability observed for the MS group in all parameters can be justified by considering that the enrolled group is heterogeneous since the EDSS score ranged from 1.5 to 4; in fact, it is already demonstrated that the changes in gait kinematics increase according to the level of disability [48]. Generally, patients with MS are also characterized by a reduced walking speed, as also demonstrated by [49].

Moving on the prolonged walking effects, the lower value of walking distance covered during the execution of the test performed by MS patients can be attributed to the fatigability effects that lead to a decrease of walking speed, shorter steps, and longer step times, as revealed by [49]. As also reported by [13], the reduced stride length may be considered as the result of a reduction of isometric quadricep strength and the consequent impossibility to produce adequate muscle moments of lower limb joints. In addition, our results confirm that the fatigue in MS patients occurs as changes in gait kinematics [25], and that the fatigue could amplifies the difference among MS patients and the control group [13] during gait.

By considering the outcomes related to δ and Δ_max_, we can affirm that the values obtained for CG are comparable with the intrinsic accuracy of the sensors [30]; for this reason, the increment/decrement occurred during the execution of the 6MWT could be due to the metrological characteristics of the adopted sensors rather than to the effects related to prolonged walking. Conversely, the decrements of the mROM and the increments of CoV and SI observed for all the patients in the MS group are greater than the sensor accuracy (see Table 7). Thus, we can likely affirm that monitoring the evolution of the here proposed indices can be an appropriate methodology for assessing the gait deterioration caused by prolonged walking.

Specifically, by focusing on the outcomes of the δ related to the ROM, the difference between CG and the most variable lower limb side of MS can be observed from the second minute only for the ankle joint, from the third minute for the knee, and only in the two last minutes for the hip. The presence of a significant decrement starting as early as first minutes at the ankle and knee level can derived to tibialis anterior, which is one of the muscles more susceptible to fatigue induced by locomotion [42] and it is one of the agonist muscles involved in the movement of knee and ankle during walking [29]. Conversely, the effects on hip joint elicited only during the last minute can be related to the capability of quadriceps to generate isometric strength longer than other lower limb muscles [50]. By combining the findings achieved in the comparison MS-CG and the ones obtained for the timing effects, we can assess that knee and ankle range of motion is early affected by the prolonged walking with respect to CG and a further deterioration occurred at the end of the task; conversely, the hip range of motion is characterized by a constant decrement that causes different values with respect to CG during the last minute. In addition, the different behavior between the most and the least variable side of MS in terms of timing effects on the range of motion and the variability confirms that prolonged walking differently affects the two lower limb sides [47]. By analyzing the gait variability, the difference between CG and the most variable lower limb side of MS can be observed from the second minute for the hip and the knee joint, while only during the last two minutes for the ankle. Thus, we can assess that prolonged walking affects differently the lower limb joint when considering the gait variability; in fact, hip joint appears to be the first one to manifest gait deterioration. Since it is already shown that greater variability may be caused by an additional activity of an antagonist [13], we can speculate that the antagonist muscles involved in the movement of hip joint generate an extra strength due to the motor coordination disrupted by the fatigue. This explanation could be reasonable since it was shown that excessive strength of the gluteus was usually produced by patients with MS also for the execution of daily life activity [50]. Regarding the effects on gait asymmetry, similar outcomes to the ROM were found; in fact, prolonged walking early affects knee joint, from the second minute, and ankle joint, from third minute; while only during the last minute at the hip joint level. Thus, we can speculate that the peripheral muscle fatigue, and more specifically the consequent altered corticospinal triggering and reduced muscle oxidative capacity [51], leads to an increase of gait asymmetry especially for the muscles involved in the movement of the knee and the ankle. Finally, the statistical differences found for all joints and all parameters related to the Δ_max_ testify that the fatigability affected both range of motion, gait variability, and gait asymmetry, causing a general gait deterioration. This effect can be ascribed to the compensatory mechanism, typical of MS when performing easy motor task that is, instead, corrupted by fatigability [13].

As concerning the correlation with the EDSS score, we can assess that the synthetic indices computed for the assessment of fatigue effects on gait asymmetry of the hip joint and the maximum difference observed for the mROM related to hip and ankle can be considered as useful tools for the quantification of clinical disability since they strongly and significantly correlated with the clinical score. This finding confirms that the evaluation of kinematic asymmetry and gait deterioration in terms of ROM variations could represent a valuable method to quantitatively measure pathology severity, as also demonstrated in other neuromuscular diseases [52].

To summarize, the results of this study clearly depict that prolonged walking causes in MS patients changes in gait patterns with respect to healthy subjects in terms of: (i) variability of range of motion; (ii) increase of gait variability; (iii) increase of gait asymmetry; and (iv) indices related to the gait asymmetry of hip and maximum difference of mROM related to hip and ankle strongly correlated with the EDSS score. In addition, differences with respect to the healthy subjects are amplified during the execution of the task [42].

As a conclusion, we want also to provide some guidelines for the implementation of specific protocols for testing the gait deterioration caused by prolonged walking. Our outcomes permit to assess that a 2-min walking test can be used to quantify the effects only if the ROM of ankle or the gait variability at the hip level are the variables of interest. As a consequence, the absence of statistical difference found in [23] in terms of blood pressure and heart rate when comparing data gathered from a 2-min walking test and a 6MWT cannot be extended also to all the kinematic parameters. Nevertheless, a shorter walking test could be asked to patients with a high level of disability, i.e., EDSS > 4, since a 6MWT could be arduous to be performed by these patients. Thus, we can speculate that the selection of the walking time of the experimental protocol should be a trade-off between the capability of patients and the variable to examine; in fact, not one of the here examined parameters could be neglected during an instrumented gait analysis, since gait variability and gait asymmetry are shown to be independent factors in assessing gait quality [53]. In addition, the evaluation of all lower limb joints appears to be mandatory, since multiple sclerosis differently affects them, especially in minimally impaired patients [54].

## 5. Conclusions

In this paper, we proposed synthetic indices to objectively measure the effects of prolonged walking on gait kinematics when MS patients performed prolonged walking. Proposed indices reveal themselves as reliable and repeatable both intra-day and inter-day. This fundamental metrological aspect allows assessing the evolution of range of motion, gait variability, and gait asymmetry during the execution of a 6-min walking test as useful indices for monitoring gait deterioration. In fact, results related to the evolution of kinematic parameters are comparable to the sensor accuracy only for the healthy subjects; while in patients with MS, the variability of gait parameters can be considered related to the fatigue effects since it is higher than the variability range due to the sensor accuracy. In particular, fatigue affects knee and ankle joint mainly in terms of range of motion reduction and gait asymmetry increment, whereas it affects hip joint in terms of gait variability. Moreover, gait asymmetry indices and maximum difference of mROM related to hip and ankle strongly correlated with the clinical disability score. Outcomes should be taken into account when designing specific treatments to reduce fatigue phenomenon.

## Figures and Tables

**Table 1:** Demographic details of enrolled patients © 2019 IEEE.

ID	Age	Height [m]	Mass [kg]	Sex	Diagnosis Date	Disease Stage	EDSS Score
ID1	44	1.65	62	F	2017	Remission	3
ID2	36	1.55	48	F	2015	Remission	1
ID3	52	1.63	73	F	1980	Remission	4
ID4	33	1.60	63	F	2008	Remission	1
ID5	31	1.65	59	F	2014	Remission	2
ID6	75	1.65	69	M	2016	Remission	1.5
ID7	52	1.74	69	M	2001	Remission	3.5
ID8	38	1.78	80	M	2011	Remission	1
ID9	40	1.58	62	F	1998	Remission	1.5
Mean	45	1.65	65	-	-	-	-
SD	5	0.07	9	-	-	-	-

**Table 2:** Range of the inter-class correlation coefficient (ICC) values related to the intra-day reliability analysis for synthetic indices related to the 6-min walking test (6MWT). We considered together the results for the left and right side due to the low value of symmetry index (SI) found.

	Joint	$\delta_{1-2}^{}$	$\delta_{1-3}^{}$	$\delta_{1-4}^{}$	$\delta_{1-5}^{}$	$\delta_{1-6}^{}$	Δ_max_
**mROM**	**Hip**	[0.70–0.78]	[0.71–0.80]	[0.70–0.76]	[0.71–0.83]	[0.70–0.82]	[0.75–0.91]
	**Knee**	[0.70–0.78]	[0.72–0.87]	[0.70–0.75]	[0.70–0.78]	[0.70–0.79]	[0.75–0.82]
	**Ankle**	[0.70–0.78]	[0.73–0.80]	[0.70–0.80]	[0.70–0.80]	[0.71–0.78]	[0.75–0.81]
**CoV**	**Hip**	[0.76–0.93]	[0.75–0.91]	[0.71–0.89]	[0.76–0.90]	[0.70–0.93]	[0.83–0.94]
	**Knee**	[0.75–0.90]	[0.77–0.91]	[0.71–0.95]	[0.71–0.81]	[0.73–0.93]	[0.79–0.94]
	**Ankle**	[0.76–0.91]	[0.78–0.88]	[0.74–0.85]	[0.76–0.96]	[0.73–0.90]	[0.80–0.94]
**SI**	**Hip**	[0.75–0.80]	[0.71–0.80]	[0.70–0.80]	[0.70–0.77]	[0.70–0.77]	[0.79–0.91]
	**Knee**	[0.70–0.78]	[0.70–0.73]	[0.70–0.77]	[0.70–0.75]	[0.70–0.77]	[0.81–0.89]
	**Ankle**	[0.70–0.74]	[0.72–0.77]	[0.70–0.75]	[0.71–0.73]	[0.70–0.80]	[0.80–0.91]
**WL**		[0.95–0.99]					

**Table 3:** ICC values related to the inter-day reliability analysis for synthetic indices related to the 6MWT. We considered together the results for the left and right due to the low value of SI found.

	Joint	$\delta_{1-2}^{}$	$\delta_{1-3}^{}$	$\delta_{1-4}^{}$	$\delta_{1-5}^{}$	$\delta_{1-6}^{}$	Δ_max_
**mROM**	**Hip**	0.72	0.75	0.75	0.77	0.74	0.82
	**Knee**	0.72	0.75	0.73	0.77	0.76	0.80
	**Ankle**	0.73	0.71	0.74	0.75	0.78	0.77
**CoV**	**Hip**	0.82	0.85	0.90	0.79	0.84	0.85
	**Knee**	0.85	0.89	0.85	0.93	0.83	0.85
	**Ankle**	0.83	0.90	0.86	0.86	0.89	0.90
**SI**	**Hip**	0.77	0.76	0.77	0.75	0.75	0.82
	**Knee**	0.76	0.72	0.76	0.76	0.76	0.81
	**Ankle**	0.75	0.73	0.75	0.72	0.78	0.80
**WL**		0.99					

**Table 4:** Maximum value of SD related to the intra- and inter-day repeatability analysis for synthetic indices related to the 6MWT. We considered together the results for the left and right due to the low value of SI found.

		Intra-Day						Inter-Day					
	Joint	$\delta_{1-2}^{}$	$\delta_{1-3}^{}$	$\delta_{1-4}^{}$	$\delta_{1-5}^{}$	$\delta_{1-6}^{}$	Δ_max_	$\delta_{1-2}^{}$	$\delta_{1-3}^{}$	$\delta_{1-4}^{}$	$\delta_{1-5}^{}$	$\delta_{1-6}^{}$	Δ_max_
**mROM (%)**	**Hip**	0.9	1.1	0.8	0.8	0.9	0.6	0.8	0.9	0.8	0.8	0.8	0.5
	**Knee**	1.3	1.2	1.2	1.0	0.9	0.7	1.1	0.9	1.0	0.7	0.8	0.6
	**Ankle**	1.0	0.9	1.0	0.8	0.9	0.8	0.8	0.9	0.9	0.8	0.8	0.7
**CoV (%)**	**Hip**	0.6	0.4	0.5	0.4	0.3	0.2	0.6	0.7	0.7	0.5	0.8	0.4
	**Knee**	0.6	0.5	0.7	0.5	0.4	0.5	0.9	0.6	0.4	0.7	0.8	1.0
	**Ankle**	0.7	0.4	0.4	0.6	0.4	0.3	0.9	0.5	0.7	0.8	0.8	0.9
**SI (%)**	**Hip**	1.0	1.1	0.9	0.9	1.0	0.8	0.9	0.8	0.7	0.9	0.9	0.7
	**Knee**	1.1	0.8	0.7	0.8	1.1	0.9	0.9	0.8	0.6	0.7	0.9	0.9
	**Ankle**	0.9	1.0	1.1	0.8	1.0	0.7	1.0	0.8	0.9	0.8	0.8	0.9
**WL (m)**		8.1						8.6					

**Table 5:** Mean (standard deviation) related to the walking length index and the gait speed.

	MS	CG	*p*-Value
**WL (m)**	359.0 (40.0)	572.0 (16.0)	<0.001 *
**Gait speed (m/s)**	1.59 (0.04)	0.99 (0.11)	<0.001 *

* Stands for statistical difference.

**Table 6:** Mean (standard deviation) related to the δ^r^ for the ROM and δ CoV and SI index. MS+ and control group (CG) represent the most variable side of patients and the control group, respectively.

		mROM (%)		CoV (%)		SI (%)	
		MS+	CG	MS+	CG	MS	CG
**Hip**	$\delta_{1-2}$	1.6 (3.4)	0.8 (1.5)	0.7 (0.5) *	0.3 (0.4) *	1.6 (1.6)	0.7 (0.6)
	$\delta_{1-3}$	2.2 (5.0)	1.4 (1.1)	0.8 (0.4) *	0.4 (0.2) *	1.8 (2.1)	0.8 (0.5)
	$\delta_{1-4}$	1.9 (5.5)	1.2 (1.0)	1.0 (0.4) *	0.4 (0.2) *	2.5 (2.2)	0.9 (0.7)
	$\delta_{1-5}$	4.5 (2.4) *	1.4 (0.9) *	1.0 (0.3) *	0.4 (0.2) *	2.2 (2.2)	1.0 (0.8)
	$\delta_{1-6}$	4.9 (2.2) *	1.2 (0.8) *	1.2 (0.5) *	0.4 (0.3) *	2.5 (2.0) *	0.8 (0.8) *
	**Δmax**	4.8(1.5) *	1.9 (0.8) *	1.7 (0.3) *	0.8 (0.2) *	4.1 (1.1) *	1.2 (0.6) *
**Knee**	$\delta_{1-2}$	1.4 (1.9)	0.6 (0.7)	1.2 (0.7) *	0.4 (0.3) *	1.9 (1.3) *	0.9 (0.5) *
	$\delta_{1-3}$	2.2 (1.4) *	0.2 (0.8) *	1.3 (0.8) *	0.5 (0.3) *	2.3 (1.6) *	0.6 (0.5) *
	$\delta_{1-4}$	1.8 (1.5) *	0.1 (0.5) *	1.5 (0.8) *	0.4 (0.2) *	2.4 (1.4) *	0.7 (0.6) *
	$\delta_{1-5}$	2.7 (2.0) *	0.1 (0.9) *	1.7 (1.0) *	0.3 (0.2) *	2.6 (1.3) *	0.7 (0.7) *
	$\delta_{1-6}$	4.5 (1.9) *	0.2 (0.9) *	2.3 (1.0) *	0.3 (0.3) *	2.8 (1.4) *	0.8 (0.6) *
	**Δmax**	4.5 (1.4) *	1.8 (0.7) *	2.4 (0.6)	0.8 (0.3)	3.8 (1.0) *	1.0 (0.4) *
**Ankle**	$\delta_{1-2}$	3.9 (2.1) *	0.4 (1.0) *	0.6 (0.7)	0.4 (0.3)	2.0 (2.6)	1.4 (0.9)
	$\delta_{1-3}$	3.5 (2.2) *	0.1 (0.5) *	0.9 (0.4)	0.5 (0.3)	2.8 (3.1)	1.6 (1.1)
	$\delta_{1-4}$	4.7 (1.9) *	0.2 (0.7) *	1.3 (0.6) *	0.3 (0.2) *	3.2 (3.3) *	1.5 (1.1) *
	$\delta_{1-5}$	6.4 (2.9) *	0.5 (0.9) *	1.5 (0.8) *	0.3 (0.2) *	4.2 (5.2) *	1.4 (1.0) *
	$\delta_{1-6}$	7.7 (2.2) *	0.6 (1.0) *	2.2 (0.9) *	0.4 (0.3) *	4.9 (5.4) *	1.2 (0.9) *
	**Δmax**	6.9 (2.4) *	2.1(1.0) *	2.3(0.5) *	0.4 (0.3) *	7.2 (3.8) *	2.9 (0.4) *

* Indicates statistical difference between group.

**Table 7:** Mean (standard deviation) related to the δ^r^ for the ROM and δ CoV and SI index. MS+ and MS− represent the most and the least variable side of patients, respectively.

		mROM (%)		CoV (%)		SI (%)
		MS+	MS−	MS+	MS−	MS
**Hip**	$\delta_{1-2}$	1.6 (3.4)	1.4 (2.1)	0.7 (0.5)	0.7 (0.4)	1.6 (1.6)
	$\delta_{1-3}$	2.2 (5.0)	2.3 (2.5)	0.8 (0.4)	1.0 (0.6)	1.8 (2.1)
	$\delta_{1-4}$	1.9 (5.5)	2.4 (2.7)	1.0 (0.4)	1.3 (0.6)	2.5 (2.2)
	$\delta_{1-5}$	4.5 (2.4)	3.3 (1.9)	1.0 (0.3)	1.0 (0.6)	2.2 (2.2)
	$\delta_{1-6}$	4.9 (2.2)	3.9 (2.1)	1.2 (0.5)	1.8 (0.5)	2.5 (2.0)
**Knee**	$\delta_{1-2}$	1.4 (1.9)	0.8 (1.0)	1.2 (0.7)	0.3 (0.2)	1.9 (1.3)
	$\delta_{1-3}$	2.2 (1.4)	1.2 (0.7)	1.3 (0.8)	0.8 (0.5)	2.3 (1.6)
	$\delta_{1-4}$	1.8 (1.5)	1.0 (1.4)	1.5 (0.8)	1.1 (0.9)	2.4 (1.4)
	$\delta_{1-5}$	2.7 (2.0)	1.1 (1.1)	1.7 (1.0)	1.4 (0.8)	2.6 (1.3)
	$\delta_{1-6}$	4.5 (1.9)	1.8 (1.3)	2.3 (1.0)	2.0 (0.8)	2.8 (1.4)
**Ankle**	$\delta_{1-2}$	3.9 (2.1)	2.7 (2.6)	0.6 (0.7)	0.7 (0.6)	2.0 (2.6)
	$\delta_{1-3}$	3.5 (2.2)	2.3 (3.1)	0.9 (0.4)	0.7 (0.5)	2.8 (3.1)
	$\delta_{1-4}$	4.7 (1.9)	3.8 (3.1)	1.3 (0.6)	1.1 (0.4)	3.2 (3.3)
	$\delta_{1-5}$	6.4 (2.9)	3.8 (2.9)	1.5 (0.8)	1.7 (0.5)	4.2 (5.2)
	$\delta_{1-6}$	7.7 (2.2)	5.3 (2.9)	2.2 (0.9)	1.9 (0.4)	4.9 (5.4)

**Table 8:** Correlation coefficient (adjusted *p*-value) related to the correlation between expanded disability status scale (EDSS) and each of the computed parameter for the most and variable side of patients. Gray cells indicate strong and significant correlation.

		mROM	CoV	SI
**Hip**	$\delta_{1-2}$	0.21 (0.72)	0.12 (0.22)	0.90 (<0.01)
	$\delta_{1-3}$	0.32 (0.81)	0.17 (0.66)	0.65 (0.04)
	$\delta_{1-4}$	0.25 (0.41)	0.24 (0.52)	0.72 (<0.01)
	$\delta_{1-5}$	0.19 (0.67)	0.36 (0.52)	0.88(<0.01)
	$\delta_{1-6}$	0.32 (0.74)	0.15 (0.70)	0.81 (0.01)
	**Δ_max_**	0.70 (0.01)	0.21 (0.71)	0.82 (<0.01)
**Knee**	$\delta_{1-2}$	0.51 (0.44)	0.63 (0.09)	0.45 (0.22)
	$\delta_{1-3}$	0.42 (0.57)	0.27 (0.57)	0.54 (0.12)
	$\delta_{1-4}$	0.23 (0.12)	0.26 (0.49)	0.23 (0.84)
	$\delta_{1-5}$	0.34 (0.15)	0.31 (0.78)	0.21 (0.57)
	$\delta_{1-6}$	0.42 (0.77)	0.29 (0.48)	0.37 (0.12)
	**Δ_max_**	−0.61 (0.08)	0.32 (0.86)	0.42 (0.34)
**Ankle**	$\delta_{1-2}$	0.55 (0.21)	0.15 (0.29)	0.32 (0.46)
	$\delta_{1-3}$	0.12 (0.42)	0.37 (0.09)	0.31 (0.22)
	$\delta_{1-4}$	0.19 (0.44)	0.15 (0.91)	0.89 (<0.01)
	$\delta_{1-5}$	0.27 (0.43)	0.48 (0.70)	0.15 (0.69)
	$\delta_{1-6}$	0.15 (0.24)	0.33 (0.29)	0.22 (0.44)
	**Δ_max_**	0.70 (0.02)	0.25 (0.84)	0.41 (0.27)
